# Immediate Dentin Sealing in Indirect Restorations: Biological Rationale, Clinical Protocol, and Contemporary Evidence

**DOI:** 10.7759/cureus.117395

**Published:** 2026-10-03

**Authors:** Sruthi Sakaray, Vanika Jain, Alisha Jain, Roshini Ravula, Pranavi Chandra Karrothi, Jerrish Joseph Antony

**Affiliations:** 1 Department of Dentistry, Rajiv Gandhi University of Health Sciences, Karnataka, IND; 2 Department of Dentistry, Baba Jaswant Singh Dental College Hospital and Research Institute, Ludhiana, IND; 3 Department of Dentistry, Pacific Medical University, Rajasthan, IND; 4 Department of Dentistry, Government Dental College and Hospital, Vijayawada, IND; 5 Department of Dentistry, St Joseph Dental College, Eluru, IND; 6 Department of Dentistry, Jagadguru Sri Shivarathreeshwara (JSS) Dental College and Hospital, Mysore, IND

**Keywords:** bond strength, dentin bonding, hybrid layer, immediate dentin sealing, indirect restorations, postoperative sensitivity

## Abstract

Immediate dentin sealing (IDS) is a newly developed adhesive procedure that involves sealing of freshly exposed dentin's surface immediately after preparation and before fabrication and placement of an indirect restoration. Nowadays, the technique has become more and more popular in modern adhesive dentistry, which attempts to achieve optimum dentin bonding in ideal clinical conditions. IDS promotes hybrid layer formation and, when applied before provisionalization, may enhance the quality and longevity of the dentin-resin bond. The current evidence suggests that IDS can improve the bond between dentin and indirect restorations and may help to decrease dentin hypersensitivity after restoration placement. A sealed dentin surface can also offer protection against contamination by bacteria, mechanical irritations, and other conditions that may be met during the provisional phase. Also, IDS can help with better adaptation of indirect restorations and more predictable resin cementation by maintaining the integrity of the adhesive interface. Clinical issues, such as choosing an adhesive system, handling an oxygen-inhibited layer, ensuring proper polymerization, and avoiding buildup of too much adhesive, are critical in order to have predictable results. While there are differences in clinical protocols and long-term evidence is somewhat mixed, the available literature is encouraging IDS as a biologically valid and clinically viable approach for indirect adhesive restorations. In conclusion, IDS is a useful tool in current restorative dentistry, especially when achieving high dentin bond strength, reducing post-preparation sensitivity, and preserving the intact dentin surface is paramount.

## Introduction and background

Immediate dentin sealing (IDS) is the immediate application of an adhesive resin to freshly prepared dentin, before impression fabrication, provisionalization, and/or placement of the indirect definitive restoration [1]. This technique is now receiving a great deal of attention in modern adhesive dentistry, as recently cut dentin is relatively clean, uncontaminated, and moist, making it a desirable substrate for an adhesive bond. Delayed sealing of dentin after the provisional phase, however, can leave the dentin surface susceptible to potential contamination from saliva, temporary cements, plaque, and other materials, which can have a negative impact on subsequent dentin adhesion [2]. The IDS concept is to seal the dentin as soon as possible after the preparation to create a strong and stable resin-dentin interface before the onset of the clinical demands of provisionalization and definitive cementation [3].

One of the major benefits of IDS from a biological standpoint is that exposed dentinal tubules are immediately occluded. In tooth preparation, the enamel will be removed and expose dentin, which may lead to an increase in dentinal permeability and hence fluid movement through the dentinal tubules [4]. This fluid movement is related to dentinal sensitivity, especially when the prepared tooth is exposed to thermal, mechanical, or osmotic stimuli. Once the dentin is prepared, the use of an adhesive layer shortly protects it from dentin permeability by sealing the dentinal tubules and consequently decreases the movement of fluids through the dentin substrate [5]. IDS can therefore potentially lead to better patient comfort during provisionalization and can minimize postoperative sensitivity after placement of the definitive restoration.

Beyond its mechanical role, the polymerized resin layer formed during IDS may also limit the oral elution of unreacted monomers and bisphenol A degradation products from the restorative interface, though the long-term biocompatibility of these coatings still warrants investigation [6]. Depending on the adhesive system and restorative protocol, the IDS surface may require cleaning, surface refreshing, or reactivation before definitive cementation; this generally involves removal of contaminants, controlled surface preparation, and application of an appropriate reactivation treatment before resin cementation of the definitive restoration.

For the clinical success of an IDS, not only the right selection of the adhesive is important but also the management of the sealed dentin surface during impression making, provisional cementation, and final cementation. The choice of the adhesive system must be based on the type of restorative material, type of cementation strategy, and properties of the prepared substrate [7]. Adequate isolation and contamination control are important since contamination of the sealed surface can affect the subsequent bonding operation. Depending on the adhesive system and restorative protocol to be used, the IDS surface might need to be cleaned, surface refreshed, or reactivated before definitive cementation [8].

IDS is a biological, adhesive, and indirect restorative dentistry approach in general. The purpose of the technique is to maximize the initial bond between the resin and dentin, to plug dentinal tubules, to decrease dentin permeability and sensitivity, and to provide good marginal and interfacial adaptation [9]. The indications for IDS are based on the biological rationale and the current evidence, and although clinical outcomes may be influenced by the adhesive system, restorative material, cementation procedure, and the operator's technique, IDS is a helpful strategy to increase the predictability of adhesive indirect restorations [10].

This narrative review aims to summarize the biological rationale, adhesive strategies, clinical protocol, and contemporary evidence supporting IDS in indirect restorations. Particular emphasis is placed on the selection of adhesive systems, management of the sealed dentin surface during provisionalization, surface reactivation before definitive cementation, and the effects of IDS on bond strength, marginal integrity, postoperative sensitivity, and pulpal protection. Although several systematic reviews and meta-analyses have evaluated specific aspects of IDS, the available evidence remains heterogeneous with respect to adhesive systems, restorative materials, cementation strategies, and clinical protocols. Therefore, this review integrates the biological principles and practical clinical steps of IDS with current evidence to provide a clinically oriented overview. The subsequent sections describe the principal adhesive systems, the sequential clinical protocol, and the reported effects of IDS on restorative and biological outcomes.

## Review

Literature search and review approach

This narrative review was developed to synthesize the biological rationale, adhesive strategies, clinical protocol, and contemporary evidence concerning IDS in indirect restorations. Relevant literature was identified through searches of PubMed/MEDLINE, Scopus, Web of Science, and Google Scholar using combinations of the terms “immediate dentin sealing”, “IDS”, “dentin sealing”, “indirect restorations”, “adhesive cementation”, “resin-dentin bond”, “postoperative sensitivity”, “marginal adaptation”, and “pulpal response.” Recent systematic reviews, meta-analyses, clinical studies, in vitro investigations, and seminal publications were considered according to their relevance to the scope of the review. The literature search primarily emphasized contemporary evidence while retaining foundational studies necessary to explain the development and biological basis of IDS. As this was a narrative review, no formal PRISMA-based screening, quantitative meta-analysis, or a formal risk-of-bias assessment was performed.

Adhesive systems and materials used in IDS

IDS is an indirect restorative technique that consists of an adhesive procedure in which the freshly prepared dentin is sealed immediately after tooth preparation before the provisional stage and the impression stage. This method is to (1) prevent contamination of the etched dentin surface and (2) create the adhesive interface in a controlled manner. The bond strength with better formation of gaps, reduced bacterial leakage, and reduced dentin sensitivity in indirect bonded restorations have been reported with IDS [11]. 

The composition and interaction between the adhesive system and the tooth substrate are responsible for dentin bonding. The modern dental adhesives typically consist of resin monomers, initiators, inhibitors/stabilizers, solvents, and, in some cases, inorganic fillers. The handling properties of the adhesive and its response to enamel and dentin will be specific to the composition, so the selection of the material is an important consideration for predictable adhesive procedures (Table 1) [12].

**Table 1:** Comparison of adhesive systems used for immediate dentin sealing IDS, immediate dentin sealing.

Adhesive system	Main clinical steps	Advantages for IDS	Limitations/considerations
Three-step etch-and-rinse	Phosphoric acid etching → primer → adhesive resin	Predictable dentin infiltration; established reference technique; strong and durable resin-dentin interface	Technique-sensitive; requires careful moisture control; multiple clinical steps [12]
Two-step etch-and-rinse	Phosphoric acid etching → combined primer/adhesive	Fewer steps than three-step systems; effective dentin bonding	Greater technique sensitivity than simplified systems; solvent evaporation is important [13]
Two-step self-etch	Self-etch primer → adhesive resin	Does not require separate phosphoric acid etching of dentin; reduced risk of postoperative sensitivity	Smear layer is incorporated rather than completely removed; enamel bonding may require selective etching [14]
One-step self-etch	Combined conditioning, priming and bonding	Simplified application; reduced clinical steps	Greater variability in bonding performance; generally less favorable for demanding adhesive procedures [15]
Universal adhesive	Self-etch, etch-and-rinse or selective enamel etching approach	Versatile; allows modification of the etching strategy according to substrate	Performance varies among formulations; selective enamel etching may be advantageous when enamel bonding is critical [16]

The definitive cementation approach should be selected according to the substrate and restorative material; adhesive resin cementation is particularly relevant for adhesive ceramic and resin-based indirect restorations, whereas self-adhesive or conventional cementation may be considered when the restorative material and preparation provide appropriate indications.

Three-step etch-and-rinse adhesives are still a significant consideration as a reference technique for dentin bonding. It includes the steps of phosphoric acid etching, primer, and adhesive resin placement. The smear layer will be modified or removed by etching, exposing the underlying dentin substrate, and the primer and adhesive will allow for the infiltration of the dentin by resin and the creation of the hybridized interface. The interface between the adhesive and dentin is an important aspect of the restoration and therefore must be carefully controlled to ensure a stable interface [13].

To simplify the bonding process, self-etch adhesive systems were developed that include both substrate conditioning and priming. Self-etch systems do not completely remove the smear layer but rather interact with and utilize parts of the smear layer in the bonding interface. They are effective by virtue of their adhesive acidity, composition, and action with dentin and enamel. Another challenge for self-etch systems may be that there are more of them in the mild category, and those can also be more challenging to bond to enamel, as a result of which the clinical bonding approach must be chosen based on the substrate and restorative circumstances [14].

Modern adhesive systems also contain universal adhesives, which can be used with various etching methods. They can be used as a self-etch, etch-and-rinse, or selective enamel etching technique, depending on the clinical context. Adhesive strategy has been shown to have an effect on the performance of the restoration, and differences have been found in the performance of various materials in a single adhesive class. When self-etch techniques are employed in cases where reliable enamel bonding is needed, selective enamel etching is an important factor to consider [15]. The use of an adhesive, filled adhesive resins, and low-viscosity resin coatings can assist with clinical handling of IDS and aid in the development of a continuous adhesive interface. It is important to maintain the integrity of the adhesive/dentin interface because flaws or failure at this interface can affect the performance of the overlying restoration [16]. The main clinical justification of IDS is that freshly prepared dentin may be conditioned and sealed before exposure to contamination that occurs when taking impressions, using provisional restorations, saliva, or temporary cements. The conditions at the adhesive interface at this stage could facilitate the conditions of bonding during later adhesive cementation. A systematic review and meta-analysis showed that IDS is a technique that may be effective at improving bond strength under experimental conditions [17]. Proper clinical execution is still important to a successful IDS. The prepared dentin should be properly isolated and treated as per the chosen adhesive system, and the sealed surface should be protected during the provisional phase. Provisional materials and temporary cements should be handled in such a way that they do not interfere with the subsequent adhesive cementation. The choice of bonding method should also follow the manufacturer's guidelines and the properties of the substrate [18].

The development of dental adhesive materials has continued to focus on controlling adhesive composition and enhancing the durability of the adhesive interface. The interaction between the adhesive and dental tissues can be affected by changes in the formulation components, such as the monomer system, solvents, and fillers. From a clinical point of view, the class and technique used for an adhesive can impact the performance of the restorative material [19]. The overall concept is a rational approach to protecting freshly prepared dentin and setting the adhesive interface before moving on to provisional and cementation procedures of indirect restoration. It is effective when proper case selection, the correct adhesive selection, substrates are managed properly, and clinical application is carefully performed. In the present scenario, some evidence supports using IDS for adhesive cementation of indirect restorations, especially in a controlled clinical environment [20].

Clinical protocol for IDS

The principle of IDS is that an adhesive sealant is best applied immediately after preparation of the tooth to seal the freshly exposed dentin surface before contamination. At this stage, dentin sealing ensures a good penetration of the resin into the collagen matrix, which will lead to the creation of a stable hybrid zone and a long-lasting bonding between the resin and dentin, thus playing a crucial role in the success of indirect restorations [21].

The IDS protocol starts with conservative preparation following the principles of minimally invasive dentistry, which aims to maintain the sound tooth structure. After preparation, the enamel surface is exposed to 37% phosphoric acid for about 2-3 seconds, allowing a good visual distinction of enamel and dentin. Etched enamel becomes a frosted appearance after rinsing and controlled air drying, and dentin has a moist, glossy appearance. The dentin is then reconditioned with a fine grit diamond or carbide bur to clean the surface and allow for the formation of an effective adhesive infiltration. When an additional resin coating is indicated, a compatible filled adhesive resin or low-viscosity flowable resin should be selected according to the adhesive system and restorative protocol. The material should provide adequate adaptation to the sealed dentin surface without producing excessive film thickness that could interfere with the seating of the definitive restoration. The selected material should also be compatible with the subsequent resin cementation procedure and should be polymerized according to the manufacturer's recommendations. Thus, material selection should be based on the intended function of the coating, compatibility with the underlying adhesive and definitive cement, and the dimensional requirements of the indirect restoration [22].

Adhesive system is applied in several thin and even layers, where each layer is light-cured separately to ensure a complete polymerization of the adhesive and maintain the integrity of the developing hybrid layer. The thickness of dentin bonding agents (DBAs) will vary depending on the surface topography and will average around 60-80 μm on smooth convex surfaces and up to 200-300 μm on any concave surface such as a marginal chamfer [23]. The use of incremental adhesive application helps to reduce disruption of the adhesive interface and consequent generation of a more homogeneous and mechanically stable resin-dentin composite. A number of modifications have been suggested with the aim of making further improvements to the performance of IDS. Earlier studies have demonstrated that the bond interface is strengthened by the introduction of filled adhesive systems or by the application of a thin layer of flowable resin over the adhesive. The in vitro findings have shown a consistently high IDS with all indirect restorative adhesive strategies [24]. Additionally, higher bond durability has been reported if a three-step etch-and-rinse bond system is followed by an intermediate layer of flowable resin, indicating that these steps may help to enhance the long-term bond stability.

After light polymerization, DBAs usually form a superficial oxygen-inhibited layer due to oxygen quenching of the free radicals required for complete polymerization. To remove this layer, a layer of glycerin gel is placed on the adhesive surface, and another light curing cycle is performed. Glycerin acts as an oxygen barrier to allow the adhesive layer to polymerize to a greater extent, followed by a thorough water rinse or air spray [25]. Impression making is performed after the sealed preparation is lightly polished using a rubber polishing prophylaxis cup to remove any layer that might be oxygen inhibited. A separating medium (e.g., petroleum jelly) is then placed to keep provisional restorations from sticking to the adhesive-coated preparation. Selection of material in the provisional stage is also crucial in helping to achieve the integrity of the sealed dentin. Generally, silicone-based elastomeric impression materials are preferred, as they are compatible with IDS-treated surfaces, but polyether impression materials can have a negative effect on the polymerized adhesive layer. In the same way, the non-eugenol provisional cements are recommended since they limit the risk of contamination and do not interfere with subsequent adhesive bonding procedures [26].

The area to be bonded is first reactivated to regain its bonding ability before definitive cementation. Surface reactivation can be performed by simply brushing with a solution containing alcohol and a very light layer of adhesive without repeating the dentin etching process. The final indirect restoration is then cemented with a resin-based cement, with minimal cement film thickness to achieve complete seating, good marginal adaptation, and a well-integrated adhesive interface. The IDS protocol goes beyond the simple application of adhesives and includes surface preservation during the entire procedure (Table 2) [27].

**Table 2:** Clinical protocol for immediate dentin sealing

Clinical stage	Recommended procedure	Purpose
Tooth preparation	Conservative preparation with preservation of sound tooth structure	Minimize unnecessary removal of tooth tissue [22]
Substrate identification	Brief enamel etching with phosphoric acid followed by rinsing and drying	Differentiate enamel from dentin and facilitate selective enamel conditioning [23]
Dentin reconditioning	Light surface refinement using a fine-grit diamond or carbide bur when indicated	Remove surface contaminants and expose a suitable substrate for adhesive infiltration [24,25]
Adhesive application	Apply the selected dentin bonding system uniformly according to manufacturer instructions	Establish the resin-dentin adhesive interface [26]
Polymerization	Light cure each adhesive layer adequately	Promote complete polymerization and interface stability [27]
Resin coating	Apply a thin layer of filled adhesive or flowable resin when indicated	Increase the thickness and mechanical protection of the sealed interface [28]
Oxygen-inhibited layer management	Apply glycerin gel and perform additional light curing	Reduce the superficial oxygen-inhibited layer [29]
Finishing/polishing	Remove excess material and lightly polish the sealed surface	Produce a smooth, clean surface before provisionalization [30]
Provisionalization	Use an appropriate separating medium and compatible provisional materials/cements	Protect the sealed dentin without compromising subsequent bonding [31]
Definitive cementation	Reactivate the sealed surface when required and lute the restoration with resin cement	Establish the definitive adhesive connection [32]

Effect of IDS on bond strength and marginal integrity

Bond strength and marginal integrity are important parameters to assess the quality of the adhesive interface and therefore the success of indirect restorations. IDS is the sealing of freshly exposed dentin with adhesive material. This early sealing approach will prevent the dentinal fluid from flowing through the dentin, bacterial contamination, and degradation of collagen before definitive restoration [28]. In vitro studies indicate that IDS is a more robust and resilient resin-dentin interface than delayed dentin sealing. Pre-bonding before provisional restoration placement promotes resin penetration of dentin and prevents surface contamination during the provisional stage, which leads to better retention stability of the bonded restoration over time [29]. The inlays and overlays made from lithium disilicate have been evaluated in studies; the results of which indicate that the fracture resistance remains at a higher level after artificial aging in case of IDS treatment. Fracture resistance is a mechanical property but is indirectly related to the stability and durability of the underlying adhesive interface when exposed to thermal and mechanical stresses [30].

Similar results have been reported for ceramic onlays, and the samples treated with IDS showed higher resistance against fracture after thermomechanical aging. CAD/CAM ceramic restorations also show better dentin-ceramic bonding, as well as a more stable bond between the dentin and the bonded interface before and during provisionalization. All these results agree with an improvement in adhesive durability and restorative performance with IDS [31]. The evidence about marginal integrity indicates that the maintenance of a well-sealed dentin surface during the provisional phase is helpful to prevent marginal gap formation and maintain tooth-restoration adaptation before final cementation, as the result of using IDS [32].

Further enhancements have been emphasized when used with biomodification processes such as application of proanthocyanidin. These strategies can help to maintain the integrity of the dentinal collagen network and minimize degradation of the hybrid layer over time, which can lead to more stable and durable resin-dentin bond [33]. A systematic review also showed that reinforced IDS protocols (with flowable resin coatings) might be better in terms of stress distribution and adhesive behavior. Further well-designed long-term clinical studies are warranted to address the variability in outcomes associated with different adhesive systems, restorative materials, and experimental designs (Table 3) [34].

**Table 3:** Effect of IDS compared with delayed dentin sealing IDS, immediate dentin sealing.

Clinical/experimental outcome	Immediate dentin sealing	Delayed dentin sealing	Overall interpretation
Resin-dentin bond strength	Generally higher	Generally lower	IDS provides an opportunity for controlled adhesive infiltration before contamination [29]
Dentin contamination	Reduced because dentin is sealed immediately	Greater potential for contamination during provisionalization	Favors IDS [28]
Marginal integrity	Generally improved	Greater susceptibility to marginal defects	Favors IDS [31,32]
Fracture resistance	Increased in several aged indirect restoration models	Comparatively lower in several studies	Potential mechanical benefit of IDS [30]
Adhesive interface durability	Potentially improved	More vulnerable to contamination and interfacial degradation	Favors IDS [33-35]
Postoperative sensitivity	May be reduced	May be greater in some clinical situations	Evidence remains inconsistent [36,37]
Pulpal protection	Potential benefit through reduction of dentinal permeability and contamination	Less immediate protection of exposed dentin	Biologically favors IDS, although clinical evidence varies [35,38-40]
Clinical sensitivity to technique	Requires careful isolation, adhesive application, and provisional management	Fewer dedicated sealing steps	IDS requires strict protocol adherence [26,27]

Effect of IDS on postoperative sensitivity and pulpal response

IDS is the process of sealing the freshly cut dentin surface with a DBA at the time of the preparation. It results in an early hybrid layer that functions as a barrier to the dentin tubules and lowers the dentin permeability. Limiting fluid movement in dentinal tubules, based on hydrodynamic theory, can be useful to reduce postoperative sensitivity [35].

There is conflicting evidence on postoperative sensitivity. Some studies have shown that IDS decreases dentinal fluid movement and creates a barrier during the provisional stage, which may reduce dentinal hypersensitivity [36,37]. Systematic reviews have shown low-certainty evidence and no statistically significant difference in postoperative sensitivity between dentin sealing and delayed dentin sealing [38,39]. These differing results may be due to the variability in clinical procedures, adhesive systems, and evaluation techniques.

IDS has generally been a good thing with regard to pulpal response. The sealing of dentin immediately after preparation will minimize bacterial contamination and microleakage between the sealing and final restorations [38]. A stable adhesive layer prevents thermal and chemical irritation, promotes pulpal health, and decreases the risk of inflammatory responses. Furthermore, IDS has been associated with increased bond strength and marginal adaptation, which lead to pulpal protection due to reduced degradation at the interface and microleakage in the long run. The direct clinical effect of IDS on postoperative sensitivity is uncertain, but there is general consensus about its beneficial effect on the biological and mechanical stability of indirect restorations and on the health of the dentin-pulp complex (Table 4) [40].

**Table 4:** Modifications intended to improve IDS performance IDS, immediate dentin sealing.

IDS modification	Proposed effect	Clinical relevance
Filled adhesive resin	Provides a thicker and potentially more resistant adhesive layer	May improve protection of the sealed dentin during provisionalization [24,34]
Flowable resin coating	Increases adhesive layer thickness and may improve stress distribution	Particularly useful in demanding indirect restorations [24,34]
Glycerin-mediated additional curing	Limits oxygen inhibition at the surface	Promotes more complete polymerization of the superficial adhesive layer [25]
Selective enamel etching	Enhances enamel conditioning while avoiding unnecessary dentin etching	Useful when universal or self-etch adhesives are used [14-16]
Surface reactivation before cementation	Restores bonding potential of the previously sealed surface	Important for achieving reliable definitive adhesion [27]
Collagen biomodification	May reduce degradation of the collagen matrix and hybrid layer	Promising strategy, but further clinical validation is required [33]

Limitations

The available evidence on IDS is heterogeneous with respect to adhesive systems, restorative materials, cementation strategies, experimental protocols, and outcome assessment methods. A substantial proportion of the available evidence is derived from in vitro investigations, which may not fully reproduce the biological, mechanical, and clinical conditions encountered in vivo. Clinical studies also differ in follow-up duration and methods used to assess postoperative sensitivity, marginal integrity, and restoration survival. In addition, differences in surface treatment and reactivation protocols may contribute to variability in reported outcomes. Therefore, although the available evidence generally supports the biological rationale and potential clinical benefits of IDS, the findings should be interpreted in the context of these methodological differences, and further well-designed long-term clinical studies are warranted.

## Conclusions

IDS is a simple yet effective modification of the conventional adhesive protocols that is in harmony with the concepts of minimally invasive, biologically driven dentistry. IDS can effectively seal freshly prepared dentin, achieving a predictable seal, minimizing dentin permeability and postoperative sensitivity, enhancing stress distribution and marginal adaptation, and maintaining a stable dentin substrate for definitive bonding. Although careful clinical execution is important, existing evidence shows that IDS is a reliable and conservative treatment that can improve the biological protection and long-term success of indirect adhesive restorations.

## References

[1] Magne P (2005). Immediate dentin sealing: a fundamental procedure for indirect bonded restorations. J Esthet Restor Dent.

[2] Magne P, Kim TH, Cascione D, Donovan TE (2005). Immediate dentin sealing improves bond strength of indirect restorations. J Prosthet Dent.

[3] Magne P, So WS, Cascione D (2007). Immediate dentin sealing supports delayed restoration placement. J Prosthet Dent.

[4] Pashley DH, Pashley EL, Carvalho RM, Tay FR (2002). The effects of dentin permeability on restorative dentistry. Dent Clin North Am.

[5] Chunara F, Dehankar SP, Sonawane AA, Kulkarni V, Bhatti E, Samal D, Kashwani R (2025). Advancements in biocompatible polymer-based nanomaterials for restorative dentistry: exploring innovations and clinical applications: a literature review. Afr J Biomed Res.

[6] Lahiri I, Roy P, Jindal A (2026). Influence of organic reinforcements on the structural and mechanical properties of epoxy dental composites. Cureus.

[7] Hardan L, Devoto W, Bourgi R (2022). Immediate dentin sealing for adhesive cementation of indirect restorations: a systematic review and meta-analysis. Gels.

[8] van den Breemer C, Özcan M, Cune MS, Ayres AA, Van Meerbeek B, Gresnigt M (2019). Effect of immediate dentin sealing and surface conditioning on the microtensile bond strength of resin-based composite to dentin. Oper Dent.

[9] Samartzi TK, Papalexopoulos D, Sarafianou A, Kourtis S (2021). Immediate dentin sealing: a literature review. Clin Cosmet Investig Dent.

[10] Alghauli MA, Alqutaibi AY, Borzangy S (2025). Clinical benefits of immediate dentin sealing: a systematic review and meta-analysis. J Prosthet Dent.

[11] Ozer F, Batu Eken Z, Hao J, Tuloglu N, Blatz MB (2024). Effect of immediate dentin sealing on the bonding performance of indirect restorations: a systematic review. Biomimetics.

[12] Breschi L, Maravic T, Mazzitelli C (2025). The evolution of adhesive dentistry: from etch-and-rinse to universal bonding systems. Dent Mater.

[13] Pashley DH, Tay FR, Breschi L, Tjäderhane L, Carvalho RM, Carrilho M, Tezvergil-Mutluay A (2011). State of the art etch-and-rinse adhesives. Dent Mater.

[14] Van Meerbeek B, Yoshihara K, Yoshida Y, Mine A, De Munck J, Van Landuyt KL (2011). State of the art of self-etch adhesives. Dent Mater.

[15] Cuevas-Suárez CE, da Rosa WL, Lund RG, da Silva AF, Piva E (2019). Bonding performance of universal adhesives: an updated systematic review and meta-analysis. J Adhes Dent.

[16] Nikaido T, Takahashi R, Ariyoshi M, Sadr A, Tagami J (2012). Protection and reinforcement of tooth structures by dental coating materials. Coatings.

[17] Antoniou I, Mourouzis P, Dionysopoulos D, Pandoleon P, Tolidis K (2024). Influence of immediate dentin sealing on bond strength of resin-based CAD/CAM restoratives to dentin: a systematic review of in vitro studies. Biomimetics.

[18] Takimoto M, Ishii R, Iino M (2012). Influence of temporary cement contamination on the surface free energy and dentine bond strength of self-adhesive cements. J Dent.

[19] Breschi L, Mazzoni A, Ruggeri A, Cadenaro M, Di Lenarda R, De Stefano Dorigo E (2008). Dental adhesion review: aging and stability of the bonded interface. Dent Mater.

[20] van den Breemer CR, Cune MS, Özcan M, Naves LZ, Kerdijk W, Gresnigt MM (2019). Randomized clinical trial on the survival of lithium disilicate posterior partial restorations bonded using immediate or delayed dentin sealing after 3 years of function. J Dent.

[21] Tjäderhane L, Nascimento FD, Breschi L (2013). Strategies to prevent hydrolytic degradation of the hybrid layer-a review. Dent Mater.

[22] Swift EJ Jr (2009). Critical appraisal: immediate dentin sealing for indirect bonded restorations. J Esthet Restor Dent.

[23] Spohr AM, Borges GA, Platt JA (2013). Thickness of immediate dentin sealing materials and its effect on the fracture load of a reinforced all-ceramic crown. Eur J Dent.

[24] de Carvalho MA, Lazari-Carvalho PC, Polonial IF, de Souza JB, Magne P (2021). Significance of immediate dentin sealing and flowable resin coating reinforcement for unfilled/lightly filled adhesive systems. J Esthet Restor Dent.

[25] Rueggeberg FA, Margeson DH (1990). The effect of oxygen inhibition on an unfilled/filled composite system. J Dent Res.

[26] Magne P, Nielsen B (2009). Interactions between impression materials and immediate dentin sealing. J Prosthet Dent.

[27] Mine A, Nikaido T, Matsumoto M (2021). Status of decontamination methods after using dentin adhesion inhibitors on indirect restorations: an integrative review of 19 publications. Jpn Dent Sci Rev.

[28] Elbishari H, Elsubeihi ES, Alkhoujah T, Elsubeihi HE (2021). Substantial in-vitro and emerging clinical evidence supporting immediate dentin sealing. Jpn Dent Sci Rev.

[29] Dalby R, Ellakwa A, Millar B, Martin FE (2012). Influence of immediate dentin sealing on the shear bond strength of pressed ceramic luted to dentin with self-etch resin cement. Int J Dent.

[30] van den Breemer CR, Özcan M, Cune MS, van der Giezen R, Kerdijk W, Gresnigt MM (2017). Effect of immediate dentine sealing on the fracture strength of lithium disilicate and multiphase resin composite inlay restorations. J Mech Behav Biomed Mater.

[31] Murata T, Maseki T, Nara Y (2018). Effect of immediate dentin sealing applications on bonding of CAD/CAM ceramic onlay restoration. Dent Mater J.

[32] Ashy LM, Marghalani H, Silikas N (2020). In vitro evaluation of marginal and internal adaptations of ceramic inlay restorations associated with immediate vs delayed dentin sealing techniques. Int J Prosthodont.

[33] Shafiei F, Aghaei T, Jowkar Z (2020). Effect of proanthocyanidin mediated immediate and delayed dentin sealing on the strength of premolars restored with composite resin inlay. J Clin Exp Dent.

[34] Varadan P, Balaji L, Manaswini DY, Rajan RM (2022). Reinforced immediate dentin sealing vs conventional immediate dentin sealing on adhesive behavior of indirect restorations: a systematic review. J Contemp Dent Pract.

[35] Brännström M (1986). The hydrodynamic theory of dentinal pain: sensation in preparations, caries, and the dentinal crack syndrome. J Endod.

[36] van den Breemer C, Gresnigt M, Özcan M, Kerdijk W, Cune MS (2019). Prospective randomized clinical trial on the survival of lithium disilicate posterior partial crowns bonded using immediate or delayed dentin sealing: short-term results on tooth sensitivity and patient satisfaction. Oper Dent.

[37] Josic U, Sebold M, Lins RB (2022). Does immediate dentin sealing influence postoperative sensitivity in teeth restored with indirect restorations? A systematic review and meta-analysis. J Esthet Restor Dent.

[38] Santana VB, de Alexandre RS, Rodrigues JA, Ely C, Reis AF (2016). Effects of immediate dentin sealing and pulpal pressure on resin cement bond strength and nanoleakage. Oper Dent.

[39] Nikaido T, Inoue G, Takagaki T, Takahashi R, Sadr A, Tagami J (2015). Resin coating technique for protection of pulp and increasing bonding in indirect restoration. Curr Oral Health Rep.

[40] Pérez-Soto E, Bourgi R, Hardan L (2025). Bonding to demineralized dentin: impact of immediate and delayed dentin sealing over time. Dent J.

